# Cross‐Wavelength Hierarchical Metamaterials Enabled for Trans‐Scale Molecules Detection Simultaneously

**DOI:** 10.1002/advs.202105447

**Published:** 2022-03-08

**Authors:** Yingli Wang, Benhui Dai, Chan Ma, Qi Zhang, Kang Huang, Xuan Luo, Xiangjiang Liu, Yibin Ying, Lijuan Xie

**Affiliations:** ^1^ College of Biosystems Engineering and Food Science Zhejiang University Hangzhou 310058 China; ^2^ Department of Physics Nanjing University Nanjing 210008 China; ^3^ School of Chemical Sciences The University of Auckland Auckland 1142 New Zealand

**Keywords:** metamaterials, molecular, SERS, smart sensory packaging, THz

## Abstract

Metamaterials have attracted increasing attention in sensing applications. However, the critical feature sizes of meta‐atom span several orders of magnitude in length scale, almost all the metamaterials are designed to operate at limited bands. It is challenging for a single type of meta‐atom with ultra‐broadband adaptability. Inspired by the natural hierarchical architectures, herein, the authors introduce a new constructing scheme of cross‐wavelength hierarchical metamaterials with a single type of meta‐atom that can realize enhancement of terahertz (THz) resonance and surface‐enhanced Raman scattering (SERS) at the same time. By combining multiple subwavelength structures at different hierarchical levels into a single meta‐atom, the obtained metamaterial can operate in two frequencies and realize multiple functionalities. Armed with this hierarchical metamaterial, detecting analytes as small as sub‐nanoscale chemical molecules or as big as microscale biomolecules simultaneously can be realized in one single metamaterial for the first time. As a proof‐of‐concept example, a smart sensory packaging is developed, which allowed them to real‐time monitor the kinetic growth of pathogenic bacteria and their metabolites in food without opening the packaging. They believe that their work will provide a valuable example that satisfies the unmet need for multiscale functional meta‐devices.

## Graphical abstract

A new constructing scheme of cross‐wavelength hierarchical metamaterial is presented. It consists of small structure (periodic AgNCs for localized SPR excitation at visible wavelengths) for large structure composition (periodically microstructures exhibiting resonance features at THz region), enabling simultaneous detection of large and small analytes. A smart sensory packaging is developed to demonstrate the applicability for in‐situ monitoring of analytes.

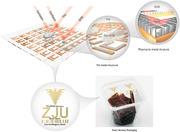

## 1 Introduction

Natural materials often show delicate hierarchical organizations and complex structures at multiple length periods and scales. The perfect combination of these structures at different hierarchical levels has endowed the living creature with many extraordinary and unusual sets of properties that usually surpass the sum of the individual components in the hierarchical architectures.^[^ ^1^, ^2^, ^3^ ^]^ The exceptional mechanical properties, such as wood and bone, are believed to be a functional adaptation of the structure at all levels of hierarchy. The insects such as moths have compound eyes with hierarchical structures, which help them to capture images with excellent motion sensitivity.^[^ ^4^ ^]^ The huge diversity of hierarchical architectures at the nano/micro scale existing in biological systems provides unlimited sources enlightening materials scientists and engineers to create next‐generation advanced functional materials.^[^ ^5^ ^]^

Instead of mimicking natural materials, metamaterials are artificial materials engineered to provide exceptional properties not found in nature.^[^ ^6^ ^]^ Metamaterials are an array of metal units (artificial meta‐atoms) structured on the subwavelength scale, giving rise to unique and exotic electromagnetic (EM) properties, such as a negative refractive index, which have attracted increasing interest for controlling EM waves. By properly designing the configuration and spatial arrangement of the meta‐atom, manipulation of EM radiation from optical frequency to THz frequency can be achieved.^[^ ^7^, ^8^, ^9^ ^]^ However, the critical feature sizes of meta‐atom spans serval orders of magnitude in length scale, from few nanometers to tens of centimeters, almost all the metamaterials are designed to operate at a specific frequency,^[^ ^10^ ^]^ a narrow band,^[^ ^11^ ^]^ or a combination of dual and triple‐band.^[^ ^12^, ^13^ ^]^ Nevertheless, once the metamaterials are fabricated, their functions will be fixed or have only a limited adjustable range. Therefore, it is challenging to fabricate metamaterials with a single type of meta‐atom with ultra‐broadband adaptability in controlling EM waves.

Inspired by the natural hierarchical architectures, herein, we propose a new constructing scheme of ultra‐broad cross‐wavelength hierarchical metamaterials. Because of the new array structure design method, two independent levels of hierarchical architectures are combined together to realize multiple frequencies’ resonances in both optical and THz frequencies and different functionalities in one single metamaterial. At the microscale hierarchical level, our metamaterial consists of a periodic array of U‐shaped meta‐atoms acting as the THz resonator. At the nanoscale hierarchical level, periodic close‐packed silver nanocube (AgNC) meta‐atoms can concentrate light on nanometer scales and generate the plasmonic “hotspot” in the gaps and corners of NC arrays, subsequently gives rise to the so‐called SERS. The unique “fingerprint” spectrum of SERS offers high sensitivity for single‐molecule detection as well as excellent adaptability for diverse targeted molecules.^[^ ^14^ ^]^ Armed with this hierarchical metamaterial, analytes as big as microscale biomolecules and as small as sub‐nanoscale chemical molecules can be detected simultaneously. As a proof‐of‐concept example, a smart packaging with sensing function was developed via our hierarchical metamaterial, which allows us to real‐time probe pathogenic bacteria and their metabolites in food simultaneously, no need to opening the packaging. We believe that our work will provide a valuable example that satisfies the practical need for multiscale functional meta‐devices, which are robust enough for real‐world applications in various fields.

## 2 Results

### 2.1 Fabrication of the Hierarchical Metamaterial

**Figure 1:**
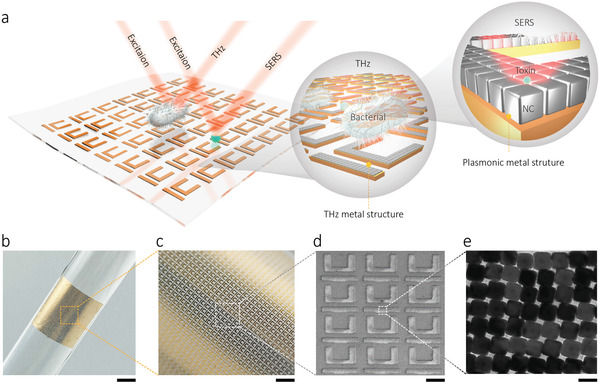
Design and hierarchical morphology characterization of the hierarchical metamaterial. a) Schematic illustration of the hierarchical metamaterial design. The metamaterial consists of four functional parts: i) a PDMS layer serves as the support layer and the stretchable layer (thickness, 80 µm); ii) a polyimide layer is the dielectric spacer, enabling the tight connection between the metallic meta‐structure and PDMS; iii) a SERS‐active plasmonic metafilm formed by an ordered AgNC superlattice, serving as sensing meta‐structure of micromolecules; and iv) the THz metasurface, which acts as a THz sensor for detecting bacteria, is made of a gold U‐shaped meta‐atoms array (thickness, 200 nm). b) Optical image of the flexible device, and c) enlarged optical image of the hierarchical metamaterial. d) SEM image of the THz metamaterial. e) High‐resolution TEM image of the SERS plasmonic metafilm. Scale bars, 2.67 mm (b), 0.70 mm (c), 93 µm (d), and 50 nm (e).

**Figure** **1**a schematically shows the hierarchical metamaterial, which consists of two levels of hierarchical architectures. At the microscale level, a periodic array of U‐shaped meta‐atoms, which can strongly confine the EM field to allow the minute detection of dielectric change, acting as the THz resonator (Figure 1b). At the nanoscale level, our metamaterial contains periodic close‐packed AgNC meta‐atoms (Figure 1c), which can enhance local electric fields via the excitation of the localized surface plasmon resonance (LSPR). This leads to a phenomenon known as SERS effect, which is a highly sensitive optical analytical technique providing unique fingerprints—vibrational information—on specific molecules.

Figure 1b shows a photo of our hierarchical architecture, which was constructed on flexible polydimethylsiloxane (PDMS) film, attached to a glass rod. The enlarged image (Figure 1c) presents the U‐shaped resonator (UR) microstructure, which is even and uniform. A representative scanning electron microscope (SEM) image of the UR metasurface is shown in Figure 1d, indicating that very neat UR arrays with flat edges obtained by the direct laser writing method. The high‐resolution transmission electron microscopy (TEM) image (Figure 1e) confirms that a compactly arranged and highly ordered array of AgNCs ≈40 nm was formed via the Langmuir–Blodgett (LB) method (a total of 237 AgNCs were analyzed, see Figure S5, Supporting Information). The average gap size between the AgNCs was ≈1 nm (Figure S6, Supporting Information). More details on the design and fabrication of the hierarchical metamaterial are introduced in Figure S1, Supporting Information.

### 2.2 Characterization of the Hierarchical Metamaterial

**Figure 2:**
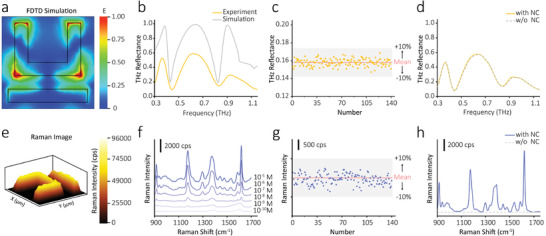
THz and SERS sensing principle of the hierarchical metamaterial. a) Simulated electric field distribution of the UR metamaterial at 0.826 THz. b) Experimental (yellow) and simulated (grey) reflectance spectra of the UR metamaterial. c) THz reflectance distribution of 140 randomly selected locations of the hierarchical metamaterial (0.826 THz). d) Experimental reflectance spectra of the UR metamaterial with/without the ordered AgNC metafilm. e) Raman intensity mapping of a unit cell of the UR metamaterial with the AgNC metafilm. f) SERS spectra of the hierarchical metamaterial detecting CV solution with different concentrations (from 0.1 nm to 10 µm with a tenfold‐increase step). g) SERS intensity distribution (≈1620 cm^−1^) of 140 randomly selected locations of the hierarchical metamaterial for CV solution (1 µm). h) SERS performance of the UR metamaterial with/without the ordered AgNC metafilm.

Finite‐difference time‐domain (FDTD) numerical simulations of the electric field of our hierarchical metamaterial were performed. **Figure** **2**a demonstrates the simulation at the microscale level. Strong electric fields are highly confined at the corner and protruding end of the U‐shaped structure at 0.826 THz, which are sensitive to the minor changes of the surrounding dielectric environment and can be utilized for sensing applications.^[^ ^15^ ^]^ The experimental resonance peaks correspond well with the simulated results (Figure 2b), where the difference in intensity is mainly due to the loss of substrate and the system parameter error between the simulation and experiment environment. As depicted in Figure 2c, the THz reflectance of 140 random locations on the hierarchical metamaterial was measured, of which the variation was less than 10% with the relative standard deviation (RSD) only ≈3.04%, indicating the uniformity of the meta‐structure and good signal reproducibility. To figure out the influence of AgNC metafilm on the THz spectral response, reflection spectra of THz metamaterials with and without AgNC metafilm were compared (Figure 2d), which turned out only 2.745 GHz shifting, suggesting that AgNC metafilm caused no influence on the THz response of the UR metamaterial. This was because the laser‐patterned AgNC metafilm just increased the thickness of the UR array, whose initial thickness had already been greater than the electron skin depth of Au in the THz region.^[^ ^16^ ^]^

To assess the SERS activity of the nanoscale hierarchical level of our metamaterial, Raman mapping measurement was implemented. As depicted in Figure 2e, the Raman imaging result presented the complete pattern of a unit cell of the UR metamaterial with high Raman signal, indicating the effective SERS effect and the even assembling of the AgNC metafilm, which suggests that the meta‐structure is of high potential for detecting trace‐level macromolecules and micromolecules simultaneously. In addition, crystal violet (CV) was used as the model analyte to evaluate the SERS performance of the hierarchical metamaterial. The observed Raman spectra are plotted in Figure 2f, and CV with a concentration of 0.1 nm can be determined by the SERS‐active meta‐structure. The SERS enhancement factor of the sensor is calculated as ≈10^7^ (see Supporting Information), which is comparable to the reported flexible plasmonic metamaterials.^[^ ^17^, ^18^, ^19^ ^]^ Moreover, the variation of Raman intensity among 140 random spots for CV solution (1 µm) on the meta‐structure was less than 10% with the RSD of ≈2.5%, indicating the reliability and good signal reproducibility of the sensor. As reported in the literature, SERS signal can be greatly increased in the inter‐nanoparticle gap (i.e., hotspots) due to the localized electric‐field enhancement.^[^ ^20^ ^]^ For the hierarchical metamaterial, a layer of AgNC metafilm can generate the SERS hotspots for sensitive Raman sensing. The simulation, using the structural parameters extracted from the high‐resolution TEM images verified it (Figure S4, Supporting Information). The electromagnetic field was increased within the gap between AgNCs, which could be used to detect molecules. The Raman enhancement effect of two metamaterials with and without NC was compared under the same concentration of CV (10^−6^ m), as shown in Figure 2h. No detectable Raman signal was obtained from the metamaterial without NC. While the substrate with NC shows a stronger Raman signal because of the electromagnetic mechanism from plasmonic AgNCs, demonstrating that the AgNC metafilm is the primary component to ensure the SERS activity of the hierarchical metamaterial. The above results proved the dual functionality of the proposed meta‐sensor and confirmed its potential in THz and Raman sensing applications.

### 2.3 Mechanical Property of the Hierarchical Metamaterial

**Figure 3:**
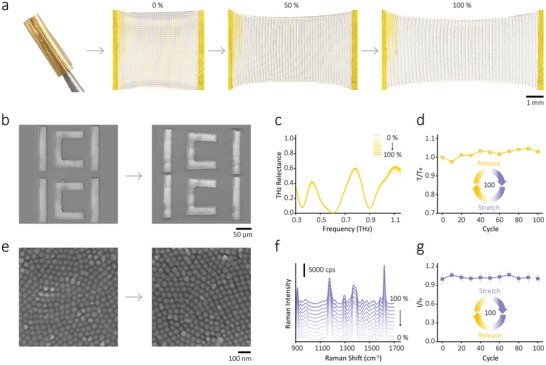
Mechanical property of the flexible hierarchical metamaterial. a) Optical images of the metamaterial under crimping and uniaxial mechanical stretching (0%, 50%, and 100%). b) SEM images of the metamaterial in its original state and 50% tensile deformation. c) Experimental THz reflectance spectra of the meta‐structure with repeated stretching and relaxation cycles (100 times). d) The THz signal change with repeated stretching and relaxation cycles (100 times). e) SEM images of the AgNC metafilm of the meta‐structure in its original state and 50% tensile deformation. f) The Raman signal of the meta‐structure under tensile deformation ranging from 0% to 100%. g) The Raman signal of the meta‐structure with repeated stretching and relaxation cycles (100 times).

For metamaterials, the mechanical property is an important feature for smart and efficient devices. Thanks to the flexible substrate layer, our metamaterial allows free bending and extensibility. The optical images of the sensor crimped on a tweezer and under tensile deformation from 0% to 100% are presented in **Figure** **3**a. It indicates that the sensor has excellent flexibility and stretchability, thus is suitable for active control of structural deformation. Figure 3b shows the SEM images of the hierarchical metamaterial in its original state and 50% tensile deformation, in which intact UR pattern is maintained and no visible cracks appear, mainly due to the adhesive effect of the thin polyimide layer. As depicted in Figure 3c, THz spectra of the metamaterial keep almost unchanged after 100 cycles of 100% deformation through a simple mechanical strain, in which the coefficient of variation during the deformation is 3.04% (Figure 3d). Moreover, the AgNCs packed tightly in both the initial and tensile state, as shown in Figure 3e, ensuring the stable SERS ability of our metamaterials. Figure 3f displays the corresponding Raman spectra under different tensile strains (0–100%), and no significant difference was found. Figure 3g shows the Raman signal of the meta‐structure with repeated stretching/relaxation cycles (100 times), in which the coefficient of variation during the deformation is 2.2%. It tells that both THz and Raman signals were not attenuated after 100 testing cycles. These results demonstrate that our flexible hierarchical metamaterial has good stability and reversibility.

### 2.4 Simultaneous Detection of Trans‐Scale Molecules

**Figure 4:**
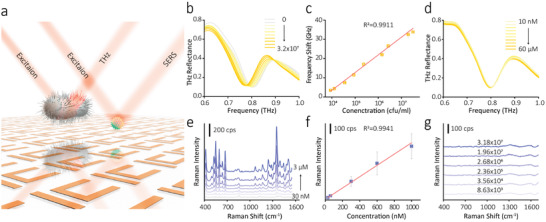
Simultaneous detection of trans‐scale molecules based on the hierarchical metamaterial. a) Schematic illustration of using the hierarchical metamaterial for detecting *P. aeruginosa* and pyocyanin simultaneously. b) THz spectra of the metamaterial with various concentrations of *P. aeruginosa*. c) The response curve of corresponding frequency shift versus bacteria concentrations. d) THz spectra of the metamaterial with various concentrations of pyocyanin. e) SERS spectra of the metamaterial detecting pyocyanin with various concentrations. f) The linear fit of SERS intensity versus the pyocyanin concentration. g) Raman spectra of the metamaterial with various concentrations of *P. aeruginosa*. The error bar indicates mean ± SD (*n* = 3).

**Figure** **4**a shows the schematic illustration of the hierarchical metamaterial comprising the nanoscale and microscale structures, which can be employed for detecting macromolecules (such as bacteria) and micromolecules (such as toxin) simultaneously. To validate this assumption, pathogenic bacteria (*Pseudomonas aeruginosa*) and their metabolites (pyocyanin) were selected as the model for this study. *P. aeruginosa*, as a kind of conditioned pathogen, can produce green water‐soluble pigment, that is, pyocyanin, which can be the auxiliary marker for identifying bacteria. In recent years, it is found that *P. aeruginosa* is the main cause of wound infection,^[^ ^21^ ^]^ which has been reported as foodborne and waterborne pathogens since its outbreak in livestock breeding and easy infection in the circulation of fruits and vegetables.^[^ ^22^ ^]^ At the microscale hierarchical level, the strong field at THz resonant response makes them highly sensitive to tiny variation in the nearby environment.^[^ ^23^ ^]^ The advantage of this kind of metamaterial is that the size of the micro‐gaps of the resonators is very similar to that of the microorganisms.^[^ ^24^ ^]^ Both of them allow for effective bacteria detection. Figure 4b shows the THz spectra containing different concentrations of *P. aeruginosa* measured by our hierarchical metamaterial, from 0 to 3.2×10^7^ cfu/mL. With the increase of bacterial concentration, a clear redshift of the resonant dip at 0.826 THz can be observed. The response curve of corresponding frequency shift versus bacteria concentration is plotted in Figure 4c. The limit of detection (LOD) was calculated to be 5000 cfu/mL. The THz spectra of detecting pyocyanin solutions with the concentration from 10 nm to 60 µm are shown in Figure 4d. No obvious frequency shift could be found. At the nanoscale hierarchical level, SERS effect excited by the AgNC metafilm generates the plasmonic “hotspot” in the gaps and corners of NC arrays. As a result of the large electric field enhancements, the possibility of a sensitive assay of trace molecules is provided.^[^ ^25^, ^26^ ^]^ Figure 4e shows the SERS spectra of detecting pyocyanin solutions from 30 nm to 3 µm. A linear relationship between the characteristic SERS intensity at 1171 cm^−1^ and the concentration was observed, which exhibits the LOD of 20 nm. The Raman spectra containing different concentrations of *P. aeruginosa* are shown in Figure 4g. The shape of the spectra is very similar, and no obvious Raman “fingerprint” appears, which shows that Raman spectroscopy cannot be used to detect *P. aeruginosa*. The above results indicate the feasibility of our hierarchical metamaterial for the simultaneous detection of trans‐scale molecules.

### 2.5 Applications as a Smart Sensory Packaging

**Figure 5:**
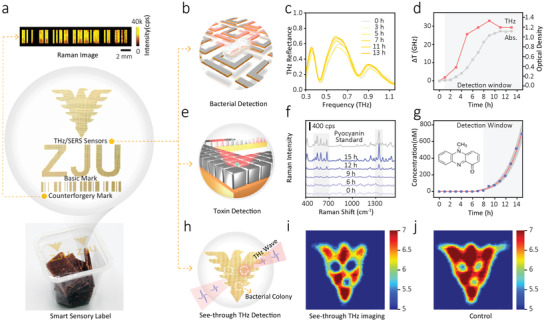
Sensing applications of the hierarchical metamaterial. a) Optical image of our hierarchical metamaterial used as the smart package label with a user‐designed mark and an anti‐counterfeiting mark. b) Schematic diagram of detecting bacteria based on the THz resonant effect. c) THz spectra of the culture medium of *P. aeruginosa* at various times based on the sensor. d) Transmittance frequency change results of THz spectrum (red line), and the growth curve of *P. aeruginosa* by measuring the absorbance of the bacterial culture at 600 nm (grey line). e) Schematic diagram of detecting toxin based on the SERS effect. f) Raman spectra of pyocyanin during different culture time. g) Real‐time monitoring of pyocyanin using the hierarchical metamaterial. h) Schematic diagram of employing the sensor as packaging material with an eagle form detecting three bacterial clusters of *P. aeruginosa*. Corresponding THz images at 0.77 THz, directly detecting (i) or detecting through food packaging (j).

To prove the function of our hierarchical metamaterial, as a proof of concept example, we developed and fabricated a sensory packaging label using the metamaterial, which can realize several functions simultaneously, such as in‐situ monitoring food safety, anti‐counterfeiting, etc. As shown in **Figure** **5**a, the sensory packaging label consisted of three functional areas. A basic mark showed the basic information of the products for the customer. An anti‐counterfeiting mark, which was obtained by dropping 10^−5^ m CV on a SERS‐active “barcode”, could only be read out by Raman spectrometer. More importantly, the label also contains a THz/SERS sensor, which can in situ monitor the *P. aeruginosa* cells and their metabolites present in the food packaging. As shown in Figure 5b, at the microscale hierarchical level, the U‐shaped arrays can be used to detect bacteria. As shown in Figure 5c, apparent redshifts were observed, and ≈1.83 GHz shift of the resonant peak at 0.826 THz was found when *P. aeruginosa* was cultured for 1 h. As the incubation time was extended to 9 h, the degree of redshift gradually increased. Next, when the incubation time was over 9 h, the red‐shift tendency was halted, and the resonant frequency remained stable after a slight blue‐shift, indicating that *P. aeruginosa* entered a stationary phase (Figure 5d). Furthermore, the growth curve of *P. aeruginosa* was monitored using an UV–visible spectroscopy, in which the absorbance at 600 nm (D_600 nm_) over 15 h was measured (Figure 5d). It showed that the growth process of *P. aeruginosa* includes a lag phase (1–4 h), exponential phage (4–9 h), and stationary phase (9–15 h). Meanwhile, at the nanoscale hierarchical level (Figure 5e), we also investigated the SERS signal of pyocyanin in the culture during the growth process of *P. aeruginosa*. The Raman fingerprint of pyocyanin began to appear at the 9th hour (Figure 5f), and the characteristic signal at 1352 cm^−1^ was significantly boosted as the incubation time went by (Figure 5g). The above results indicate the proposed hierarchical metamaterial has the capability of real‐time monitoring the kinetic growth of *P. aeruginosa* and their metabolic behaviors. This useful information will play a crucial role in future smart control, which would allow non‐destructive and real‐time monitoring of food safety and quality.

Once the feasibility of our proposed hierarchical metamaterial towards the detection of *P. aeruginosa* and pyocyanin was confirmed, we further investigated its application as smart sensing platforms in real packaged food. In real samples, food is packaged in a plastic or paper bag, so the sensor was attached inside the package bag. Additionally, with the high penetration of the THz wave and the aid of this smart sensory packaging, we can detect bacterial contamination through packaging and realize the early diagnosis of pathogenic bacteria, which is meaningful for food safety supervision and management. Figure 5h exhibits the schematic illustration of using the hierarchical metamaterial as the smart sensing label to probe the bacterial contamination of packaged food. An optical image of the sensor with an eagle showed that three bacterial clusters of *P. aeruginosa* were detected. From the results presented in Figures 5i and 5j, the bacterial colonies can be probed by THz pulses, either directly (Figure 5i) or through food packaging (Figure 5j), suggesting that it's a feasible and effective method using the sensory packaging to monitor the bacterial contamination of food in a practical package.

## 3 Discussion

Conventional metamaterials can achieve the resonant response and can be operated at a combination of dual and triple‐band.^[^ ^12^, ^27^, ^28^, ^29^, ^30^, ^31^ ^]^ They are designed for a specific application, due to the difficulty and low flexibility of adapting their functions once fabricated. Therefore, it is challenging to fabricate metamaterials with a single type of meta‐atom with ultra‐broadband adaptability in controlling EM waves, due to the huge diversity between the critical subwavelength feature sizes of the artificial atom.

Inspired by the natural hierarchical architectures, herein, we propose a new constructing scheme of cross‐wavelength metamaterials, in which several levels of hierarchical architectures combined in one single metamaterial to realize multiple frequencies’ resonances and different functionalities. Therefore, as shown in Figure 1a, one single type of meta‐atom can operate in multiple frequencies and realize different functionalities simultaneously. With this hierarchical metamaterial, analytes as big as microscale biomolecules and as small as sub‐nanoscale chemical molecules can be detected simultaneously, which is of high importance for the development of point‐of‐care devices. As these devices are dedicated to facing increasing number of detecting objects and drawing conclusions in a short time.^[^ ^32^ ^]^ Their potential applications benefit from the superiority of technologies and methods, such as plasmonic biosensing.^[^ ^33^, ^34^ ^]^ Our works of the hierarchical metamaterial are those that fabricate functional meta‐devices for multiple demands used in various fields. Furthermore, our hierarchical metamaterial also exhibits stability and reversibility properties, owing to the robust mechanical properties of the flexible substrate (as shown in Figure 3e,g), which could enable excellent flexibility and stretchability. This is an ideal configuration for developing functional devices with more practical consideration. Despite that only two different hierarchical levels were combined in this work, we believe the potential combinations are huge because the critical feature size could span seven orders of magnitude in length scale (from nanometer to centimeter) or even higher. This would promote design flexibility with frequency response. Therefore, such a constructing scheme can also help to reach high unit cell density in designing metamaterials, due to the “space‐saving” nature of the scheme. Also, such metamaterial can be adjusted by stretching to generate resonance in visible, near infrared, mid‐infrared, and THz bands, so it can achieve a span of many orders of magnitude, and realize tunable frequency selection.^[^ ^35^ ^]^ The building blocks of used metamaterial are silver nanoparticles, which tend to transform under oxic conditions. The stability test for our material was performed for a period of 16 days. The result shows that our sample is stable for up to 4 days and the signal begins to attenuate on the fifth day (Figure S7, Supporting Information). This limitation could be replaced by using other relatively stable nanoparticles^[^ ^36^, ^37^ ^]^ or adding an external shield on the surface of nanoparticles^[^ ^38^, ^39^ ^]^ to design more stable metamaterials in future studies. In this work, alternative materials including gold nanoparticles and gold film coated on ordered polystyrene beads were used to enhance the stability of the metamaterial (Figures S8 and S9, Supporting Information).

As a proof‐of‐concept example (Figure 5a), we developed a smart sensory label for practical application in food packaging, due to its excellent THz and Raman detection performance that allow simultaneous and in situ monitoring of the kinetic growth of pathogenic bacteria and their metabolites in the food matrices. Previously reported sensory labels can only monitor relatively simple parameters, such as storage temperature, humidity, orientation, freshness, or ripeness of the foods.^[^ ^40^, ^41^, ^42^, ^43^, ^44^ ^]^ However, our sensory label offers “universal” molecular recognition ability due to the unique Raman “fingerprint” spectrum. Meanwhile, the high penetration and low energy of THz ray allow it to non‐destructively “see” through the common dielectric package materials like paper and plastic, therefore enable in‐situ monitoring without opening the package (as shown in Figure 5i). We believe that our work will provide a valuable example that satisfies the practical call for multiscale functional meta‐devices that are robust enough for real‐world applications in various fields.

In conclusion, our constructing scheme of hierarchical metamaterials can bridge the existing gap in cross‐scale frequency bands, which can also be easily extended to other frequency regions besides visible and THz ranges through adjusting the size of meta‐atoms. Our work will open up a profound field, in which fascinating functions of hierarchical structures are delicately integrated to bring about more smart devices in various fields.

## 4 Experimental Section

### Fabrication Process

The hierarchical metamaterial had been fabricated starting with a bare silicon substrate as a sacrificial wafer. A layer of 1H,1H,2H,2H‐perfluorodecyl acrylate molecules was evaporated on the bottom of the Si wafer and it would act as a great intermediate layer to ease the final release process for the low bonding energy with PDMS. PDMS, acting as the stretchable layer, was spin‐coated and cured on a hot plate, followed by O_2_ plasma treatment to temporarily make PDMS hydrophilic for improving its adhesion to the subsequent layer. A polyimide solution was then sputter‐coated on the PDMS layer and the curing process of the polyimide layer was conducted at 190 °C in a convection oven for 2 h. Au (230 nm) was then evaporated on the top side of the resulting layers. The AgNCs were self‐assembled on top of the metal layer using a LB deposition, followed by defining metamaterials via laser cutting. Finally, the flexible hierarchical metamaterials were fabricated by peeling off the metamaterial layers from the Si substrate. More details about fabrication and measurements are available in the Supporting Information.

### Numerical Simulation

FDTD simulations were performed using a commercial software (Lumerical Solutions). The structural parameters for modeling were set according to the size measured from TEM images with an inter‐nanoparticle gap of 1 nm. The diameter of AgNCs was set as 43 nm, the thickness of the Au layer was defined as 230 nm according to the experimental measurements. Periodic boundary conditions were employed. The optical constants of gold and silver were employed in the package.

### Basic Experimental Set‐Up

All the THz experiments were conducted in a THz time‐domain system, which provided the broadband THz pulses in the frequency range from 0.1 THz to 3.5 THz. Raman spectra were collected using a LabRAM HR Evolution Raman microscope system equipped with an integral Olympus BX40 microscope and an automatic motorized 3D stage, employing a He—Ne laser with a power of about 17 mW for the excitation. The illumination power on the sample could be adjusted with neutral density filters inside the instrument.

### Molecular Detection by THz Spectroscopy and Raman Spectroscopy

To verify the feasibility for quantitative detection of *P. aeruginosa* and pyocyanin using this metamaterial, THz spectra and SERS spectra were recorded. For THz measurement, different concentrations of *P. aeruginosa* and pyocyanin were inactivated and centrifuged. Only 10 uL of culture medium was dripped onto the metamaterial and dried at 65 °C for ≈30 min. Then, THz reflectance spectra were recorded. For Raman measurement, SERS spectra were saved under 633 nm irradiation in the wet state after immersion of the hierarchical metamaterial in *P. aeruginosa* and pyocyanin solutions of known concentration, which exhibited a series of pyocyanin characteristic peaks for ring deformations, N‐CH_3_ wagging, ring stretching, and —CH_3_ scissoring (Figure S17, Supporting Information).

### Statistical Analysis

Data are expressed as means ± standard error of the mean and all statistical analyses were performed using OriginPro (OriginLab Corporation, USA).

## Conflict of Interest

The authors declare no conflict of interest.

## Author Contributions

Y.W. and B.D. contributed equally to this work. L.X., X.Liu, and Y.W. conceived and designed the experiments; Y.W. prepared, executed the experiments, conducted the simulations, and analyzed the data; Y.W., B.D., C.M., X.Luo, X.Liu, Y.Y., and L.X. supported experimental analyses; X.Liu, B.D., Y.Y., and L.X. prepared the manuscript; all authors contributed to data interpretation, revised the paper and approved the final manuscript.

## Supporting information

Supporting information

## Data Availability

The data that support the findings of this study are available in the supplementary material of this article.
