# Supplementary material for: Cross‐Wavelength Hierarchical Metamaterials Enabled for Trans‐Scale Molecules Detection Simultaneously
Source: Adv Sci (Weinh). 2022 Mar 8;9(13):2105447. doi: 10.1002/advs.202105447 (PMC9069183; doi:10.1002/advs.202105447)
Supplement: Supplementary file 1 — Supporting information [file ADVS-9-2105447-s001.pdf]

ADVANCED SCIENCE
Open Access

Supporting Information

for *Adv. Sci.*, DOI 10.1002/advs.202105447

Cross-Wavelength Hierarchical Metamaterials Enabled for Trans-Scale Molecules Detection Simultaneously

*Yingli Wang, Benhui Dai, Chan Ma, Qi Zhang, Kang Huang, Xuan Luo, Xiangjiang Liu, Yibin Ying and Lijuan Xie**

Supporting Information

Cross-Wavelength Hierarchical Metamaterials Enabled for Trans-scale Molecules Detection Simultaneously

*Yingli Wang#, Benhui Dai#, Chan Ma, Qi Zhang, Kang Huang, Xuan Luo, Xiangjiang Liu, Yibin Ying, Lijuan Xie**

1. Fabrication of the Hierarchical Metamaterial

1.1. Fabrication approach of the hierarchical metamaterial

As shown in Figure S1, the fabrication began with an 80-μm-thick layer of polydimethylsiloxane (PDMS) mixed at a 20:1 ratio, which was spin-coated (3000 rpm, 30 s) and cured on a hot plate at 80 ºC for 2 h. The PDMS layer acted as the stretchable layer. This was followed by an O^2 plasma treatment for 2 min, which would temporarily make PDMS hydrophilic to improve its adhesion to the subsequent layer. Subsequently, a polyimide (PI) solution was then sputter-coated on the PDMS layer and the curing process of the PI layer was conducted at 190 ºC in a convection oven for 2 h. The resulting layers was then evaporated with a 230-nm-thick Au layer as our main metal film. We self-assembled the Ag nanocube (AgNC) on top of the metal layer using a Langmuir-Blodgett deposition trough (KSV NIMA KN2002), followed by defining metamaterial by laser cutting. Finally, the flexible metamaterial was fabricated by peeling off the metamaterial layers from the silicon substrate. The geometry and dimensions are shown in Figure S1c.

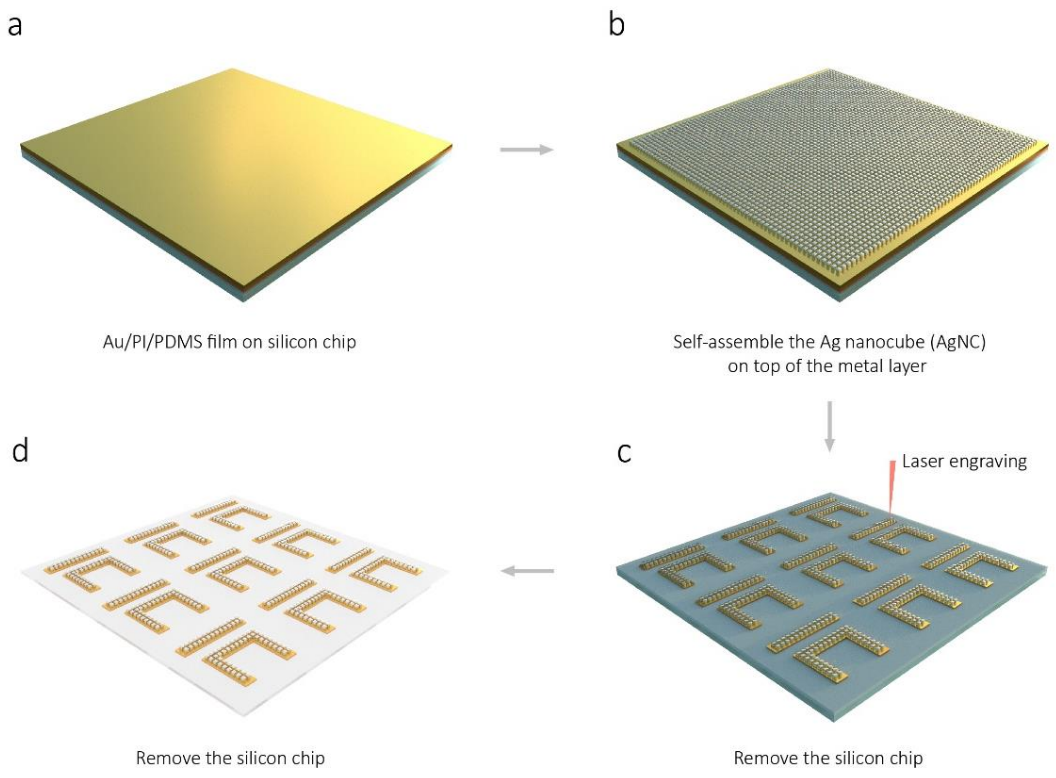

Figure S1.

Fabrication of the hierarchical metamaterial. (a) Preparing the Au/PI/PDMS film on silicon chip. (b) Ag nanocube self-assembling on top of the metal layer. (c) Defining metamaterial by laser engraving. (d) Removing the silicon chip.

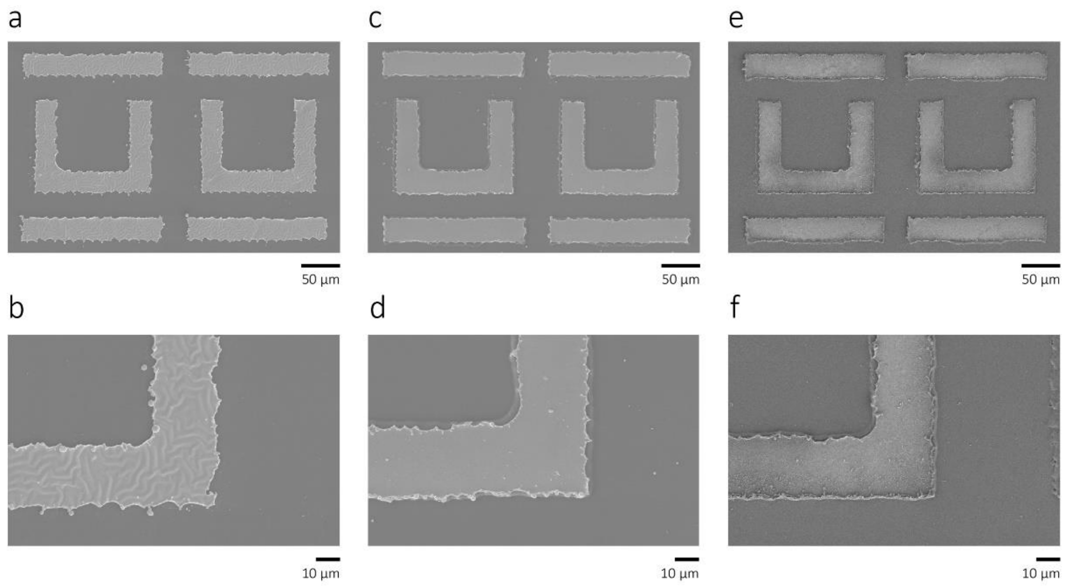

Figure S2.

SEM of the hierarchical metamaterial. (a, b) The metamaterial without PI and nanocubes. (c, d) The metamaterial without nanocubes. (e, f) The metamaterial with PI and nanocubes.

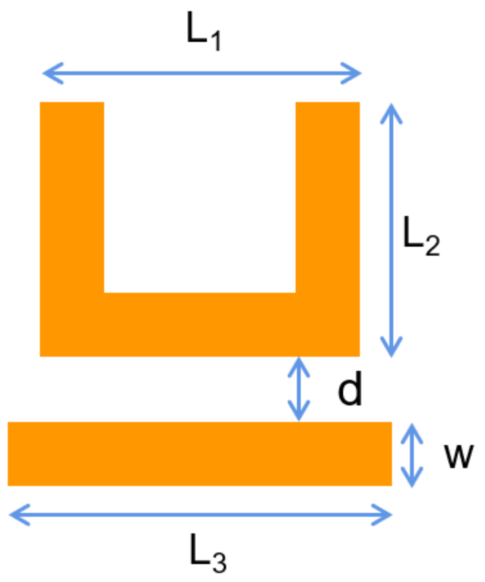

Figure S3.

The dimensions and structure of the metamaterials.

The element consists of the planar periodic array of a U-shaped structure (Figure S3). Fixing the geometry of metamaterial with L_1 = 150 µm, L_2 = 120 µm, L_3 = 180 µm, and *d* = 30 µm. The widths of all arms are fixed as *w* = 30 µm, the period of the array in vertical and horizontal directions is fixed as *P*v = *P*h = 210 µm, respectively. The dimensions of the metamaterials were estimated by scanning electron microscope (SEM) and transmission electron microscope (TEM). SEM images were collected by a field-emission scanning electron microscope. The synthesis of AgNC was prepared using seed-mediated growth method (*1*).

1.2. Estimated SERS enhancement factor (EF)

For simplicity, we used the analytical EF to estimate the SERS performance of the metamaterial, which can be calculated using the following equation:

$$EF = \frac{I_{SERS}/C_{SERS}}{I_{RS}/C_{RS}}$$

Where I_{SERS} and denotes the intensity at 1619 cm^{-1} of the Raman spectrum of crystal violet (CV) with concentration C_{SERS} in the presence of the metamaterial. I_{RS} denotes the intensity of CV with concentration C_{RS}. I_{SERS} and I_{RS} were measured under identical experimental conditions. The detailed calculation is shown below.

(1) When C_{SERS} was 1×10^{-9} M, the measured the I_{SERS} was about 536.6 counts/s.

(2) When C_{RS} was 1×10^{-2} M, the obtained I_{RS} was about 218.2 counts/s.

(3) The analytical EF = ~ 2.46×10^{7}.

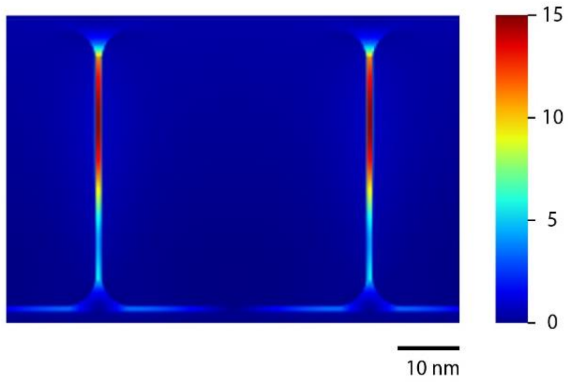

Figure S4.
FDTD simulation of the local electric field enhancement for the EM hotspot in the AgNC metasurface.

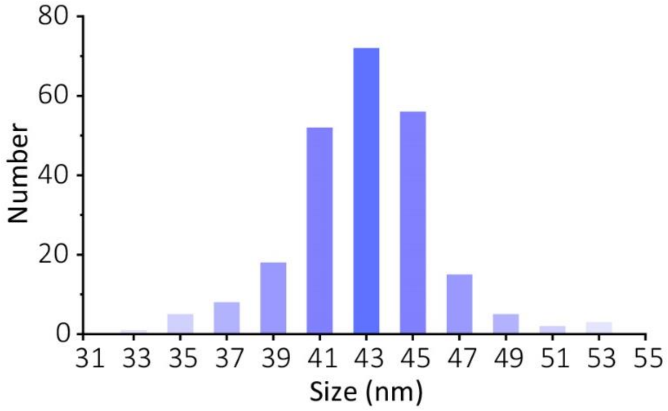

Figure S5.
Size distribution of 237 AgNCs.

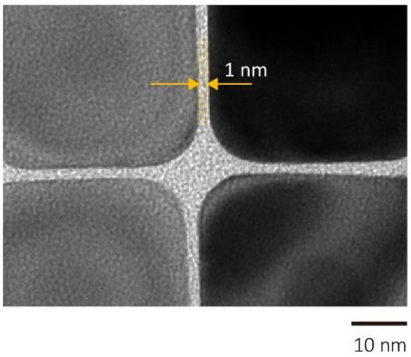

Figure S6.
High-resolution TEM image of the SERS plasmonic metafilm.

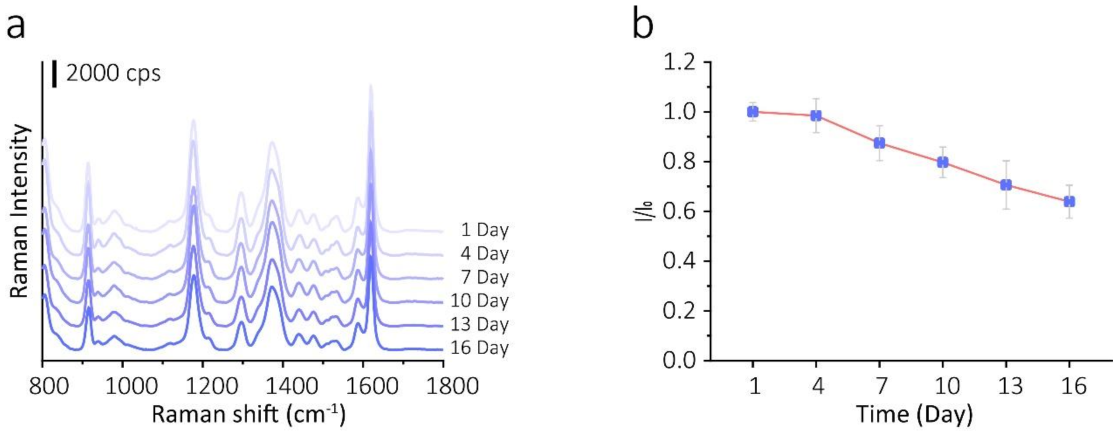

Figure S7.

Stability test. (a) SERS spectra of the silver nanoparticle metafilm detecting CV solution on different days. (b) The Raman signal of the metafilm on different days.

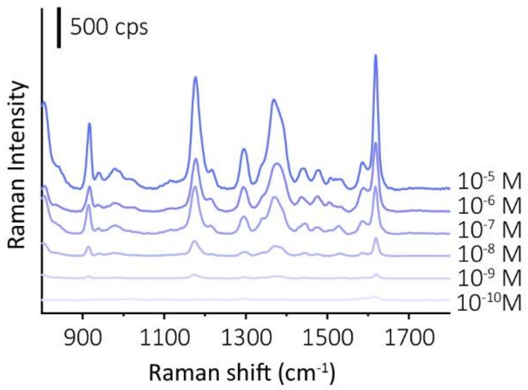

Figure S8.

SERS spectra of the gold nanoparticles metafilm detecting CV solution with different concentrations (from 0.1 nM to 10 µM with a tenfold-increase step).

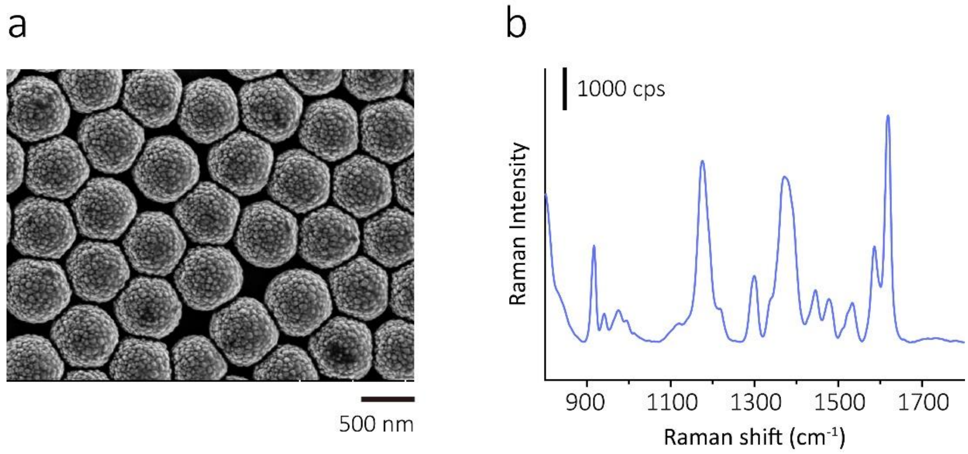

Figure S9.

SEM image of polystyrene beads (a) and SERS spectra (b) of gold film coated on ordered polystyrene beads (detecting CV solution).

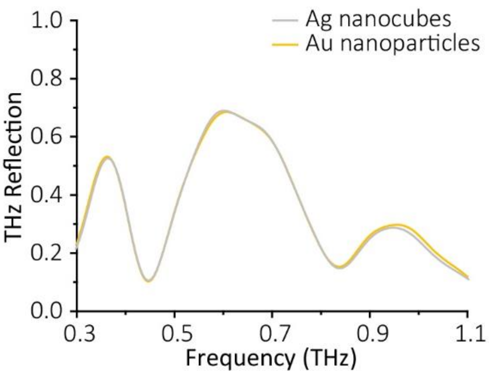

Figure S10.

THz spectra of silver nanocube and gold nanoparticle metafilm.

2. Characterization of the Device Mechanics

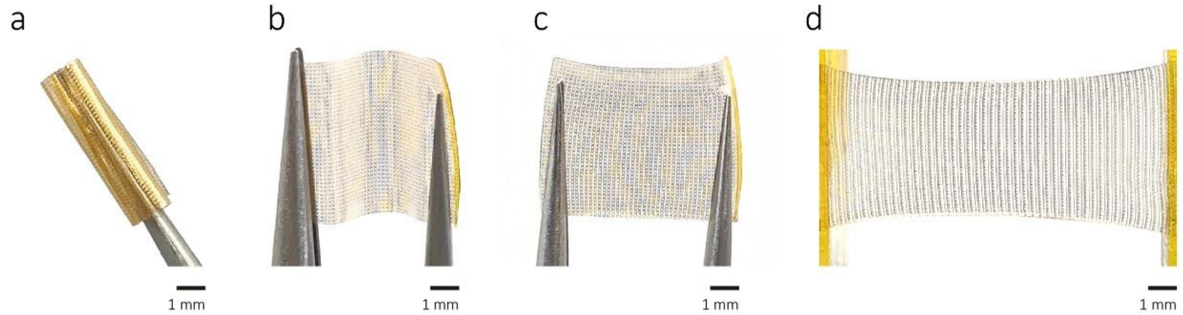

Figure S11.

Photographs of the metamaterial under various states. (a) Crimped. (b) Extruded. (c) Original. (d) Stretched. Photo Credit: Yingli Wang, Zhejiang University.

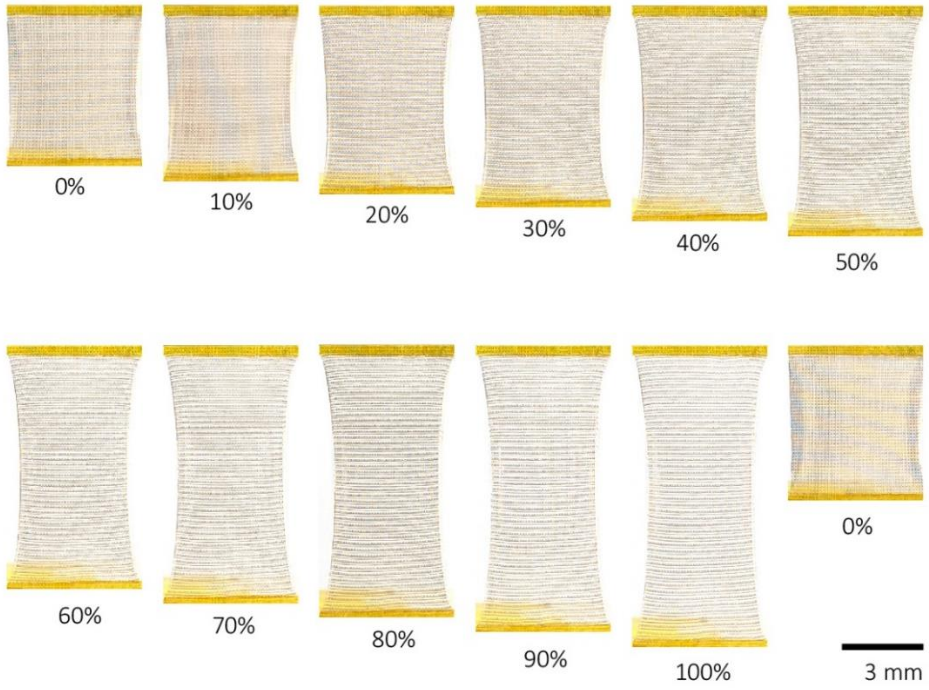

Figure S12.

Photographs of the metamaterial under various strains. Photo Credit: Yingli Wang, Zhejiang University.

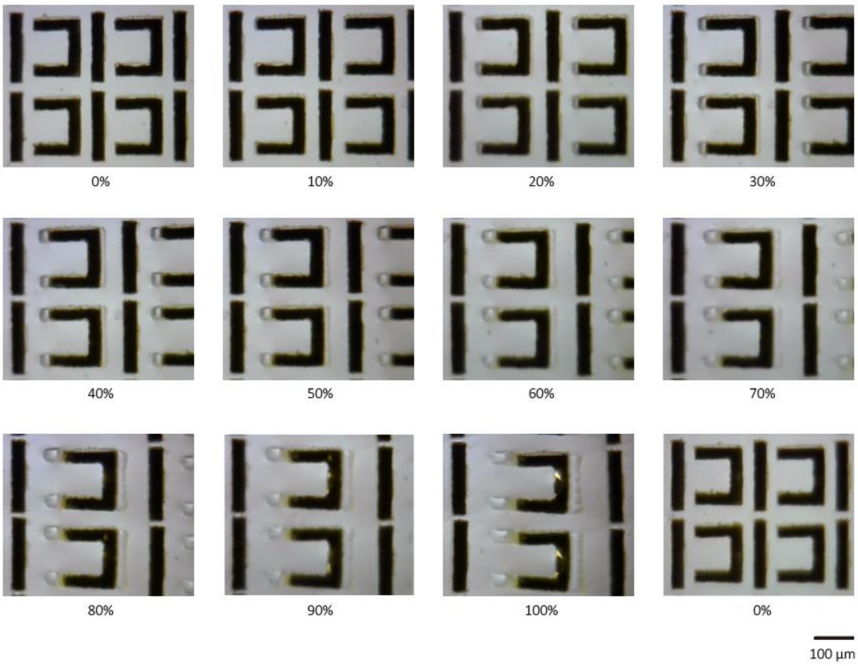

Figure S13.

Photographs of the metamaterial under various strains. Photo Credit: Yingli Wang, Zhejiang University.

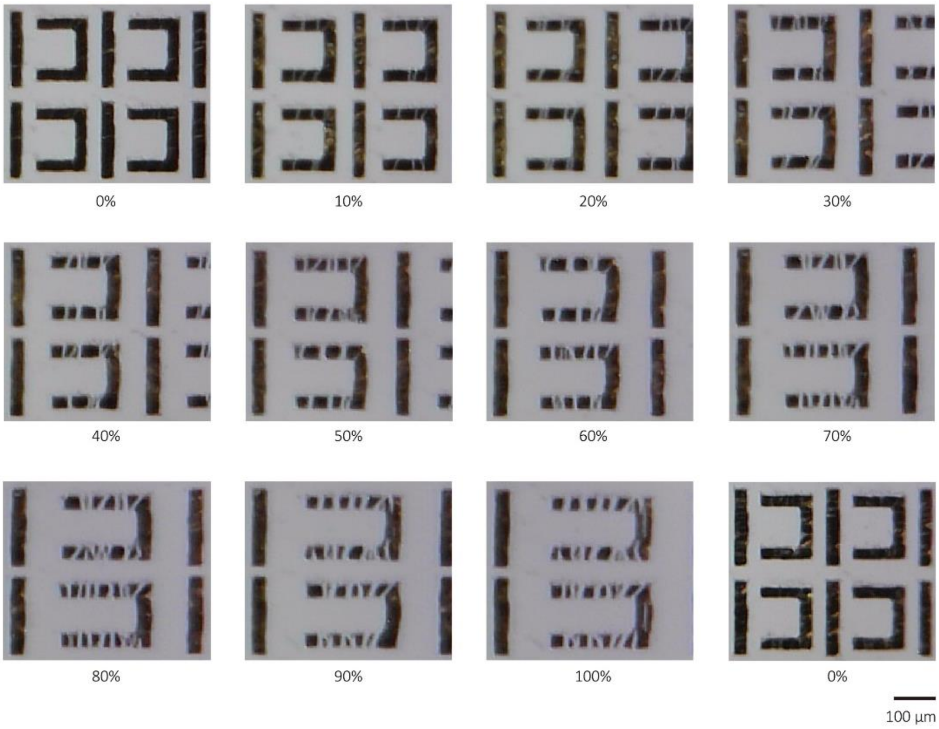

Figure S14.

Photographs of the metamaterial without PI under various strains. Photo Credit: Yingli Wang, Zhejiang University.

3. Reusability of the Metamaterial

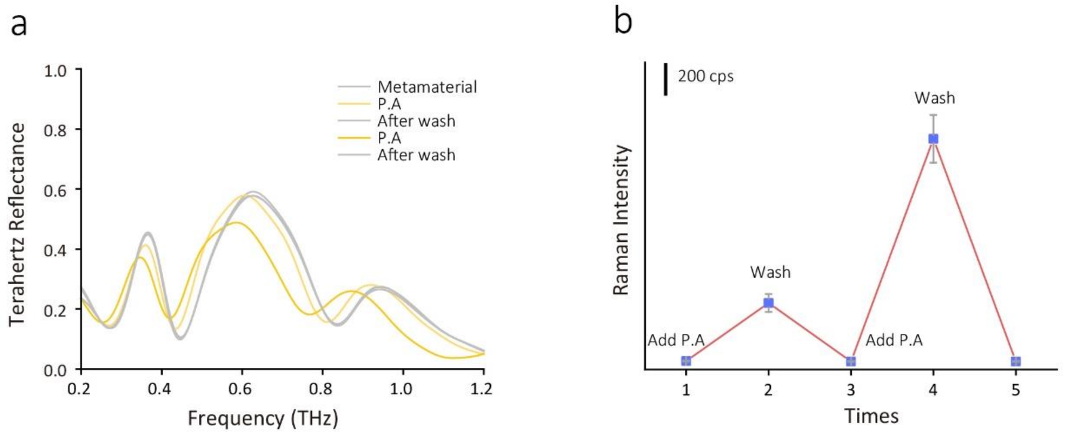

Figure S15.

Reusability of the metamaterial. (a) The reflectance THz spectra before and after using. (b) Raman spectra before and after using.

4. In-vivo Studies

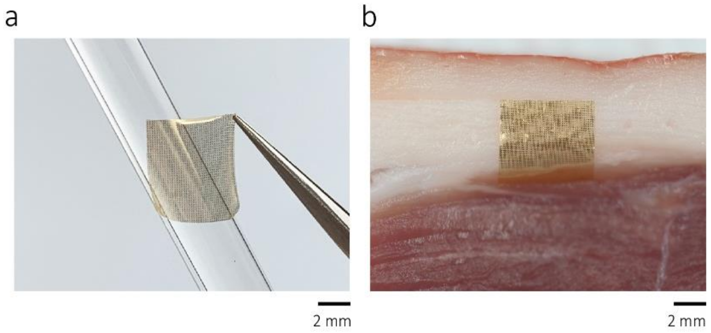

Figure S16.

Optical images of the flexible device. (a) The metamaterial stuck on the tube. (b) The metamaterial stuck on the pork. Photo Credit (all): Yingli Wang, Zhejiang University.

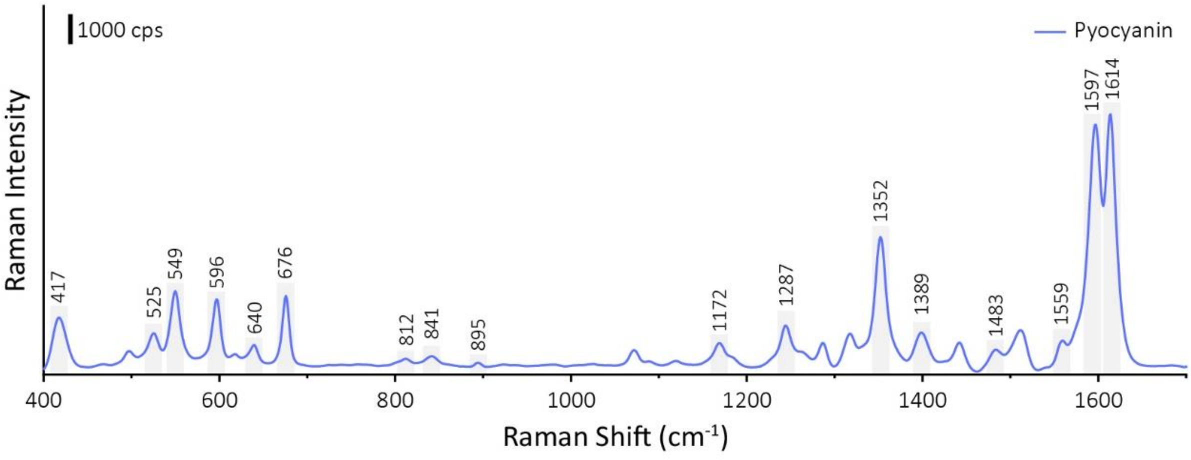

Figure S17.

Typical SERS spectrum of pyocyanin.

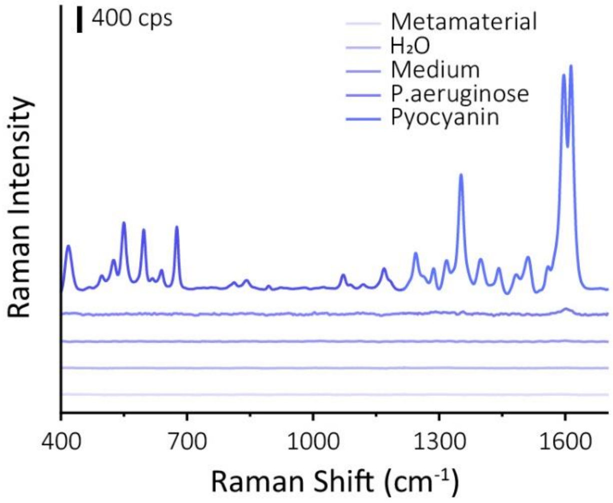

Figure S18.

Typical SERS spectra of pyocyanin and *P. aeruginosa*.

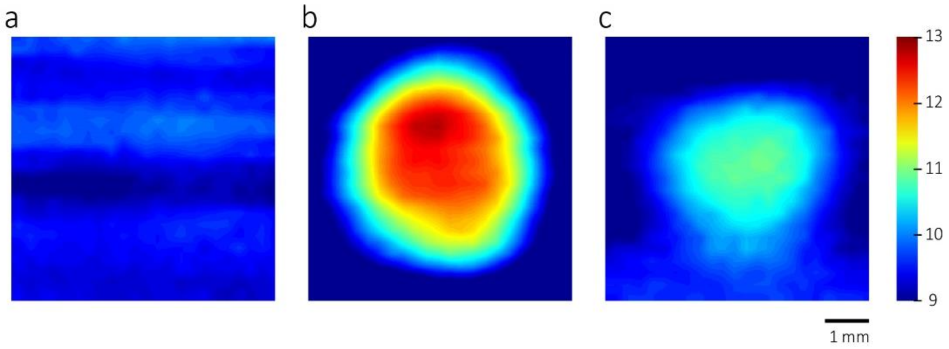

Figure S19.

Experimental imagings of the metamaterial. (a) Imaging without *P. aeruginose*. (b) Imaging dropped with *P. aeruginose*. (c) Imaging dropped with *P. aeruginosa* and PI.

References

[1] Y. Ma, W. Li, E. C. Cho, Z. Li, T. Yu, J. Zeng, Z. Xie, Y. Xia, *ACS Nano* **2021**, *4*, 6725.
